# Supplementary material for: User-defined outcomes of the Danish cardiovascular screening (DANCAVAS) trial: A post hoc analyses of a population-based, randomised controlled trial
Source: PLoS Med. 2024 May 13;21(5):e1004403. doi: 10.1371/journal.pmed.1004403 (PMC11132442; doi:10.1371/journal.pmed.1004403)

Supplementary material

**Table of contents**

[**Table A:** Definition of outcomes 2](#_Toc165663036)

[**Table B:** Primary and secondary outcomes for probably attendee within the invitation-to-screening and usual-care group 4](#_Toc165663037)

[**Table C:** Distribution of variables before and after inverse probability of treatment weighting 5](#_Toc165663038)

[**Table D:** Initiation of preventive medication 6](#_Toc165663039)

[**Table E:** Adherence to preventive medications 7](#_Toc165663040)

[**Fig A:** Survey regarding interest in a cardiovascular screening examination *- in Danish* 8](#_Toc165663041)

[**Fig B:** Hazard ratio by age of the user-defined primary outcome 10](#_Toc165663042)

[**Fig C:** Resulting ROC curve of the inverse probability of treatment weighting model 11](#_Toc165663043)

[**Fig D:** Initiation of antiplatelet (A) and lipid-lowering (B) agents during follow-up 12](#_Toc165663044)

# **Table A:** Definition of outcomes

| **Primary outcome** |  |  |
| --- | --- | --- |
| User-defined |  |  |
|  |  |  |
|  |  |  |
| **Secondary outcome** |  |  |
| - Composite outcome of major adverse cardiovascular events (MACE) | Death due to cardiovascular disease, stroke, acute myocardial infarction, heart failure assessed at December 31, 2021. | |
| - Composite outcome of major adverse limb events (MALE) | Death due to cardiovascular disease, aortic dissection and rupture, critical limb ischemia, and major amputation due to peripheral arterial disease assessed at December 31, 2021. | |
| - Causes of death (primary and participating) | Cardiovascular, cancer, other disease, and composite of trauma and suicide (time to event or censoring), assessed at December 31, 2021. | |
|  | **Diagnosis from the Danish National Patient Registry, ICD-10 codes and NOMESCO codes, assessed at December 31, 2022** | **Major vascular surgeries from the national clinical database National Vascular Registry** |
| - Stroke | I60, I61, I63, I64, I65, I66, I67, G45 |  |
| - Myocardial infarction | I21, I22 , I23, I248, I249 |  |
| - Heart failure | I500, I501, I509 |  |
| - Aortic dissection | I710 |  |
| - Aortic rupture | I711, I713, I715, I718 |  |
| - Chronic critical limb ischemia |  | Karbase=Indik_1 OR indik_2 , OR indik_3=17, 18, or 19 |
| - Amputation due to peripheral arterial disease | KNFQ09, KNFQ19, KNGQ09, KNGQ19,  KNHQ09, KNHQ19 |  |
|  |  |  |
|  |  |  |
| **Explanatory outcomes** | **Prescriptions from the Registry of Prescription Medication, ATC code, assessed at December 31, 2022** |  |
| - Antithrombotic agents | B01AC |  |
| - Anticoagulation | B01AA, B01AE, B01AF |  |
| - Lipid modifying agents | C10 |  |
| - Antihypertensive | C03A, C03B, C07, C08 excl C08DA, C09 |  |
| - Drugs used in diabetes | A10 |  |
|  | **Diagnosis from the Danish National Patient Registry, ICD-10 codes and NOMESCO codes, assessed at December 31, 2022** | **Major vascular surgeries from the national clinical database National Vascular Registry** |
| - Composite elective aortic repair | Elective thoracic aortic surgery:  FCA10, FCA30, FCA50, FCA60, FCA70,  FCB,  FCC10, FCC30, FCC50, FCC70, FCC75, FCD40, FCD50, FCD60, FCD70, FCW96  Excluding diagnosis of I710, I711, I715, I718  FCW96 14 days after I71.0 | Elective abdominal aortic surgery:  Karbase=Indik_1 OR indik_2 OR indik_3=3  AND diagnose I71.4 + I71.6 + I72.3 |
|  |  |  |

|  |  |  |
| --- | --- | --- |
| **Safety outcome** | **Diagnosis from the Danish National Patient Registry, ICD-10 codes and NOMESCO codes, assessed at December 31, 2022** | **Major vascular surgeries from the national clinical database National Vascular Registry** |
| - Major intracranial bleeding | I60, I61, I62, S064, S065, S066 |  |
| - Major gastrointestinal bleeding | K25, K26, K27, K28, K29, K625, K920, K921, K922 |  |
| - Cancer | C |  |
| - Cardiac revascularization | KFNA-E, KFNG |  |
| - Aortic repair | Thoracic aortic surgery: FCA10+30+50+60+70,  FCB, FCC10+30+50+70+75, FCD40+50+60+70 +  FCW96 | Abdominal aortic surgery:  Karbase=Indik_1 OR indik_2 OR indik_3=3, 4, 5, 8, 61, 62  AND diagnose I71.x eller I72.3 |
| - Peripheral vascular revascularization |  | Karbase=Indik_1 OR indik_2= 01, 16, 17, 18, 19 |
|  |  |  |

# **Table B:** Primary and secondary outcomes for probably attendee within the invitation-to-screening and usual-care group

|  | Events No (%) | Years at risk  Median (IQR) | No. of events per 1000 person-years | Events  No (%) | Years at risk  Median (IQR) | No. of events per 1000 person-years | Incidence risk difference (95% CI) | HR (95% CI) | p value |
| --- | --- | --- | --- | --- | --- | --- | --- | --- | --- |
|  | **Probably attendee within**  **the invitation-to-screening group** | | | **Probably attendee within**  **the usual-care group** | | |  |  | |
| **Primary outcome** | | | | | | | | | |
| Defined by the users | 1017 (6.08) | 6.58 (5.81;7.16) | 15.65 | 3420 (11.48) | 6.36 (5.62;7.08) | 17.91 | -3.96  (-5.12;-2.80) | 0.87 (0.81;0.94) | <0.001 |
| **Secondary outcomes** | | | | | | | | | |
| MACE | 1286 (7.68) | 5.59 (4.84;6.16) | 23.22 | 4810 (16.15) | 5.36 (4.66;6.08) | 27.25 | -9.11  (-10.72;-7.50) | 0.85 (0.80;0.91) | <0.001 |
| MALE | 330 (1.97) | 5.70 (4.97;6.18) | 5.69 | 1839 (6.17) | 5.55 (4.74;6.16) | 8.27 | -6.01  (-6.85;-5.16) | 0.69 (0.61;0.78) | <0.001 |
| CVD specific mortality | 194 (1.16) | 5.70 (5.08;6.18) | 3.32 | 1175 (3.94) | 5.59 (4.80;6.16) | 4.98 | -4.08  (-4.77;-3.38) | 0.67 (0.57;0.78) | <0.001 |

* The user-defined composite outcome encompassed myocardial infarction or stroke

CI; confidence interval. HR; hazard ratio. IQR; interquartile range. MACE; major adverse cardiovascular events (death due to cardiovascular disease, stroke, acute myocardial infarction, heart failure). MALE; major adverse limb events (death due to cardiovascular disease, aortic dissection and rupture, critical limb ischemia, and major amputation due to peripheral arterial disease).

Outcomes are registry based (Supplementary Table 1)

# **Table C:** Distribution of variables before and after inverse probability of treatment weighting

|  | Attenders (n=10,471) | Non-attenders (n=6,265) | Usual care (Unweighted) (n=29.790) | Usual care (weighted) (n=29.790) |
| --- | --- | --- | --- | --- |
| Age, mean (SD) | 68.71 (2.61) | 68.87 (2.70) | 68.80 (2.62) | 68.61 (2.60) |
| Prescriptions the last year before randomisation | | | | |
| - Antiplatelet agents – N (%) | 2,496 (23.8%) | 1,688 (26.9%) | 7,596 (25.5%) | 13,804 (23.4%) |
| - Anticoagulants – N (%) | 844 (8.1%) | 596 (9.5%) | 2,581 (8.7%) | 4,574 (7.8%) |
| - Lipid-lowering agents – N (%) | 3,971 (37.9%) | 2,276 (36.3%) | 11,439 (38.4%) | 24,359 (41.3%) |
| - Antihypertensive agents – N (%) | 5,340 (51.0%) | 3,401 (54.3%) | 15,763 (52.9%) | 29,925 (50.8%) |
| - Antidiabetic agents – N (%) | 1,128 (10.8%) | 1,035 (16.5%) | 3,765 (12.6%) | 4,867 (8.3%) |
| Hospital admission during the last five years before randomisation | | | | |
| - Stroke – N (%) | 405 (3.9%) | 343 (5.5%) | 1,521 (5.1%) | 2,222 (3.8%) |
| - Ischaemic heart disease* – N (%) | 405 (3.9%) | 232 (3.7%) | 1,227 (4.1%) | 2,680 (4.5%) |
| - Heart failure – N (%) | 169 (1.6%) | 227 (3.6%) | 748 (2.5%) | 708 (1.2%) |
| - PAD – N (%) | 182 (1.7%) | 191 (3.0%) | 643 (2.2%) | 783 (1.3%) |
| - Aortic aneurysms – N (%) | 149 (1.4%) | 128 (2.0%) | 449 (1.5%) | 606 (1.0%) |
| Demographic measures | | | | |
| - Living alone – N (%) | 1,596 (15.2%) | 2,113 (33.7%) | 6,904 (23.2%) | 5,581 (9.5%) |
| - Non-Danish ethnicity - N (%) | 300 (2.9%) | 341 (5.4%) | 1,235 (4.1%) | 1,218 (2.1%) |
| - Educational level |  |  |  |  |
| - 1 | 2,274 (21.7%) | 2,057 (32.8%) | 7,849 (26.3%) | 9,798 (16.6%) |
| - 2 | 5,568 (53.2%) | 3,009 (48.0%) | 15,265 (51.2%) | 32,522 (55.2%) |
| - 3 | 2,627 (25.1%) | 1,201 (19.2%) | 6,676 (22.4%) | 16,606 (28.2%) |
| - Yearly income, dkk, mean (SD) | 276,203 (347,755) | 235,975 (301,279) | 262,800 (470,559) | 312,401 (682,344) |
| - Working – N (%) | 2,961 (28.3%) | 1,327 (21.2%) | 7,577 (25.4%) | 19,338 (32.8%) |

* Ischaemic heart disease: myocardial infarction and coronary revascularization

# **Table D:** Initiation of preventive medication

|  | Events No (%) | Years at risk  Median (IQR) | No. of events per 1000 person-years | Events  No (%) | Years at risk  Median (IQR) | No. of events per 1000 person-years | Incidence risk difference (95% CI) | HR (95% CI) | p value |
| --- | --- | --- | --- | --- | --- | --- | --- | --- | --- |
|  | **Invited to screening** | | | **Usual-care group** | | |  |  | |
| Initiation of antiplatelet agents* | 4154 (24.82) | 5.08 (2.29;5.98) | 58.63 | 3382 (11.35) | 5.36 (4.66;6.08) | 22.78 | 35.84 (33.90;37.78) | 2.50 (2.39;2.62) | <0.001 |
| Initiation of anticoagulants* | 1546 (9.24) | 5.37 (4.68;6.16) | 18.11 | 2792 (9.37) | 5.37 (4.68;6.08) | 18.47 | -0.36  (-1.50;0.77) | 0.98 (0.92;1.04) | 0.54 |
| Initiation of lipid-lowering agents* | 3831 (22.89) | 5.15 (2.97;6.05) | 52.62 | 3640 (12.22) | 5.36 (4.62;6.08) | 24.74 | 27.89 (26.04;29.74) | 2.09 (1.99;2.18) | <0.001 |
| Initiation of hypertensive agents* | 2414 (14.42) | 5.27 (4.62;6.08) | 29.47 | 4247 (14.26) | 5.36 (4.62;6.08) | 29.14 | 0.33  (-1.14;1.80) | 1.01 (0.96;1.06) | 0.63 |
| Initiation of antidiabetic agents* | 822 (4.91) | 5.40 (4.74;6.16) | 9.41 | 1437 (4.82) | 5.40 (4.74;6.16) | 9.28 | 0.13  (-0.67;0.94) | 1.01 (0.93;1.11) | 0.74 |

CI; confidence interval. HR; hazard ratio. IQR; interquartile range.

* No prescription the last year before randomization.

# **Table E:** Adherence to preventive medications

|  | Non-adherent patients in invited to screening group (n(non-adherent)/n(total) (%)) | Non-adherent patients in the usual-care group (n(non-adherent)/n(total) (%)) | Relative risk of non-adherence (RR (95%CI)) |
| --- | --- | --- | --- |
| Antiplatelet agents (B01AC) | 1291/7621 (16.9) | 1392/9188 (15.2) | 1.12 (1.04;1.20) |
| Anticoagulants (B01AA, B01AE, B01AF) | 247/2266 (10 9) | 404/4029 (10.0) | 1.09 (0.94;1.26) |
| Lipid-lowering agents (C10) | 1663/9360 (17.8) | 2237/13291 (16.8) | 1.06 (1.00;1.12) |
| Antihypertensive (C03A, C03B, C07, C08 excl C08DA, C09) | 818/10104 (8.1) | 1521/18022 (8.4) | 0.96 (0.88;1.04) |

Data are n/N (%).

Adherence to a medication is defined as medication possession ratio of at least 80% from 1st redeemed prescription over a time period of 3 years. Values below 80% will be considered as non-adherence. Individuals who failed to redeem prescriptions during the first three years after randomization is not eligible for analysis, and only individuals who redeemed at least one relevant prescription are included in the analyses. The adherence results are presented as relative risks with 95% confidence intervals.

# **Fig A:** Survey regarding interest in a cardiovascular screening examination *- in Danish*


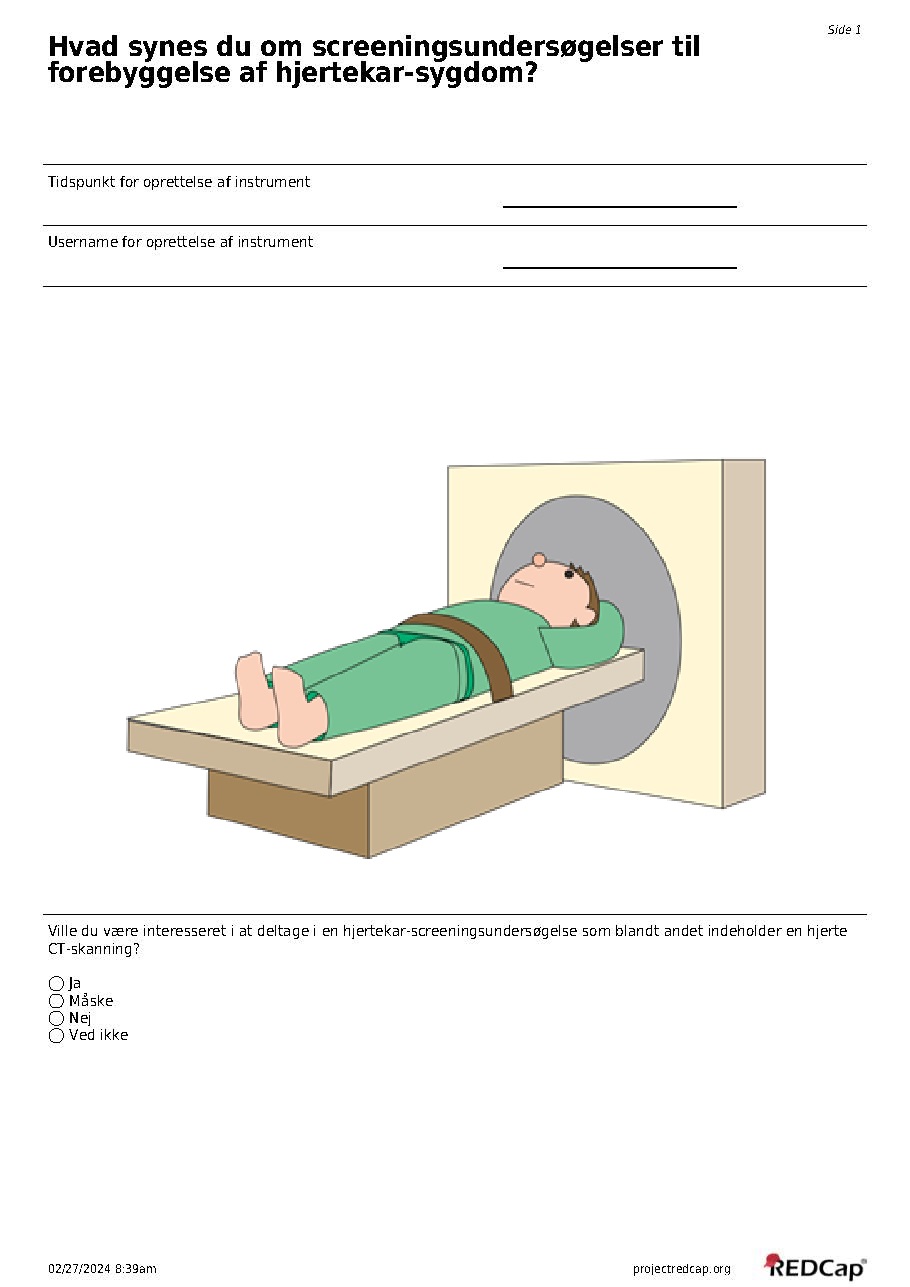


**Fig A:** Survey regarding interest in a cardiovascular screening examination *- in Danish (continued)*


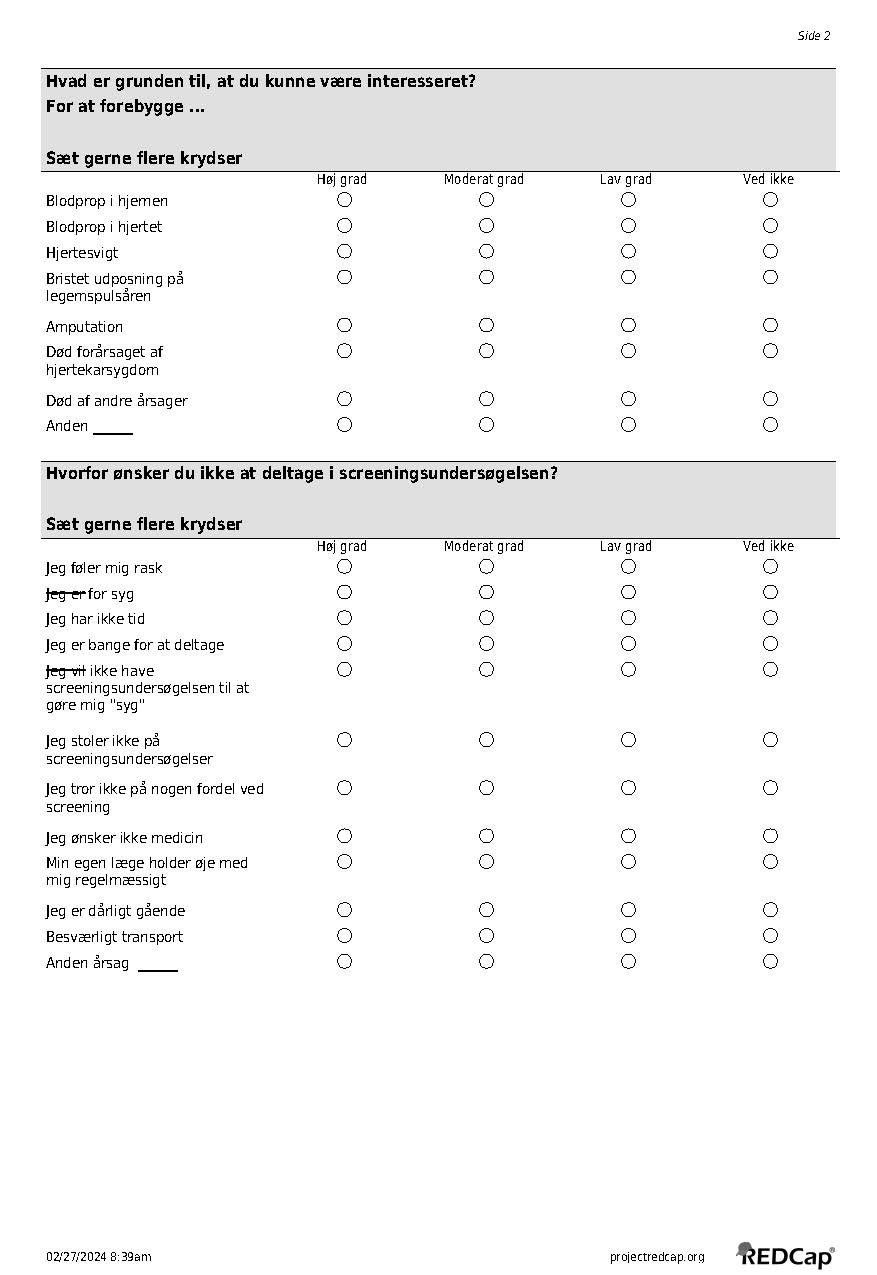


# **Fig B:** Hazard ratio by age of the user-defined primary outcome


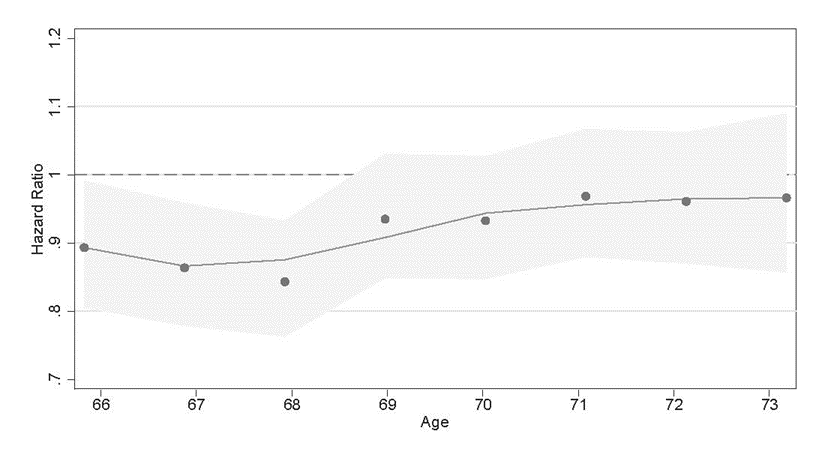


# **Fig C:** Resulting ROC curve of the inverse probability of treatment weighting model


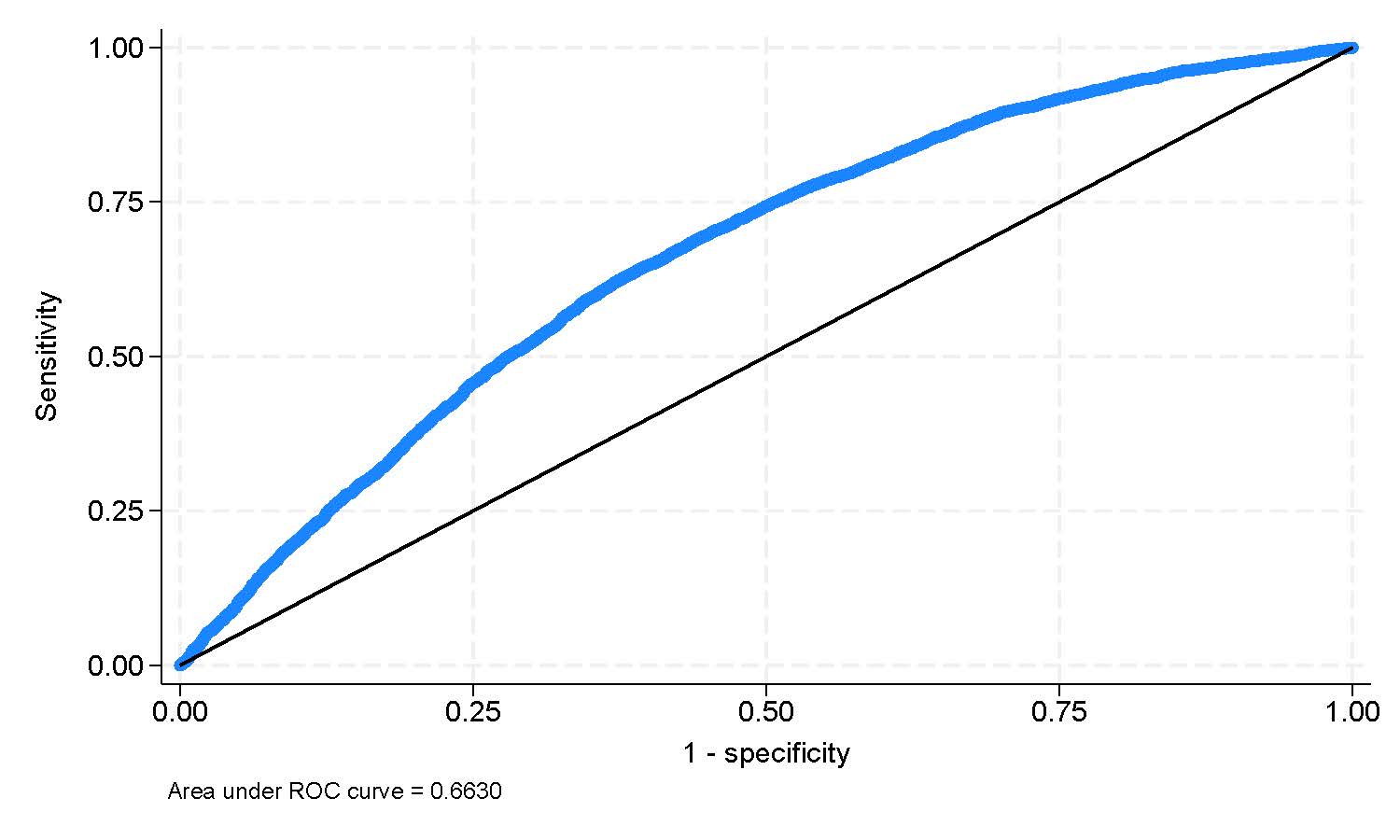


# **Fig D:** Initiation of antiplatelet (A) and lipid-lowering (B) agents during follow-up

**A**


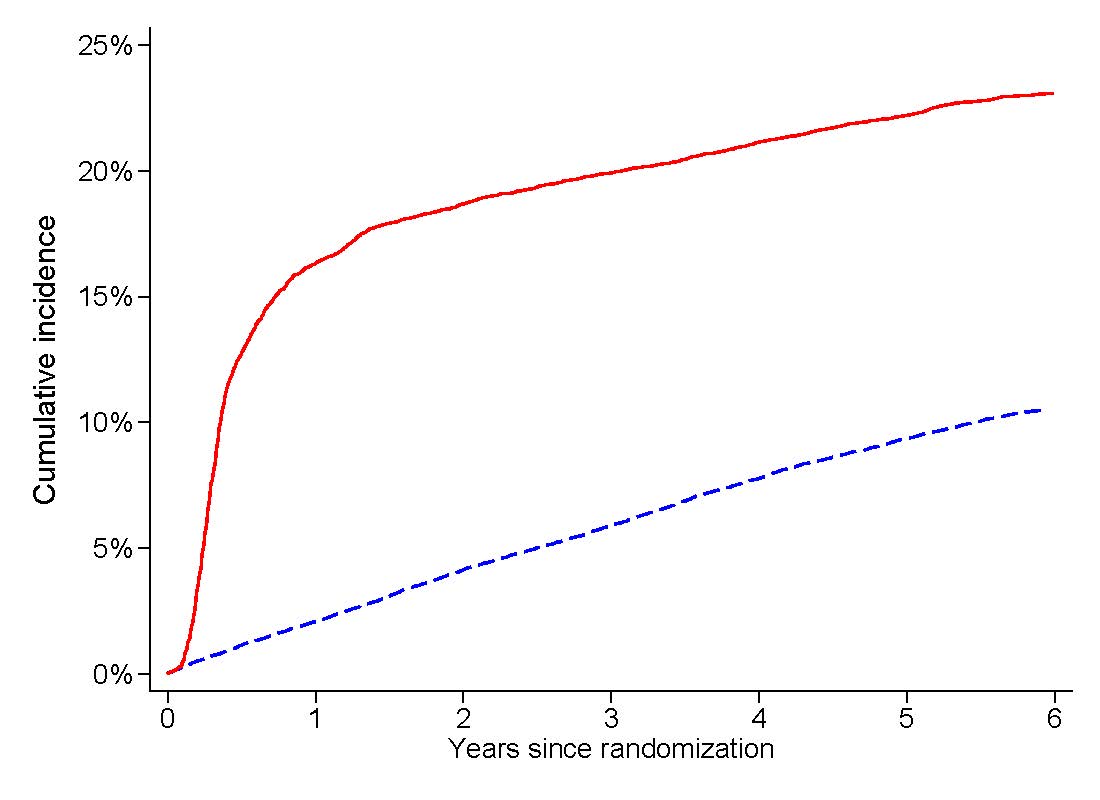


**B**


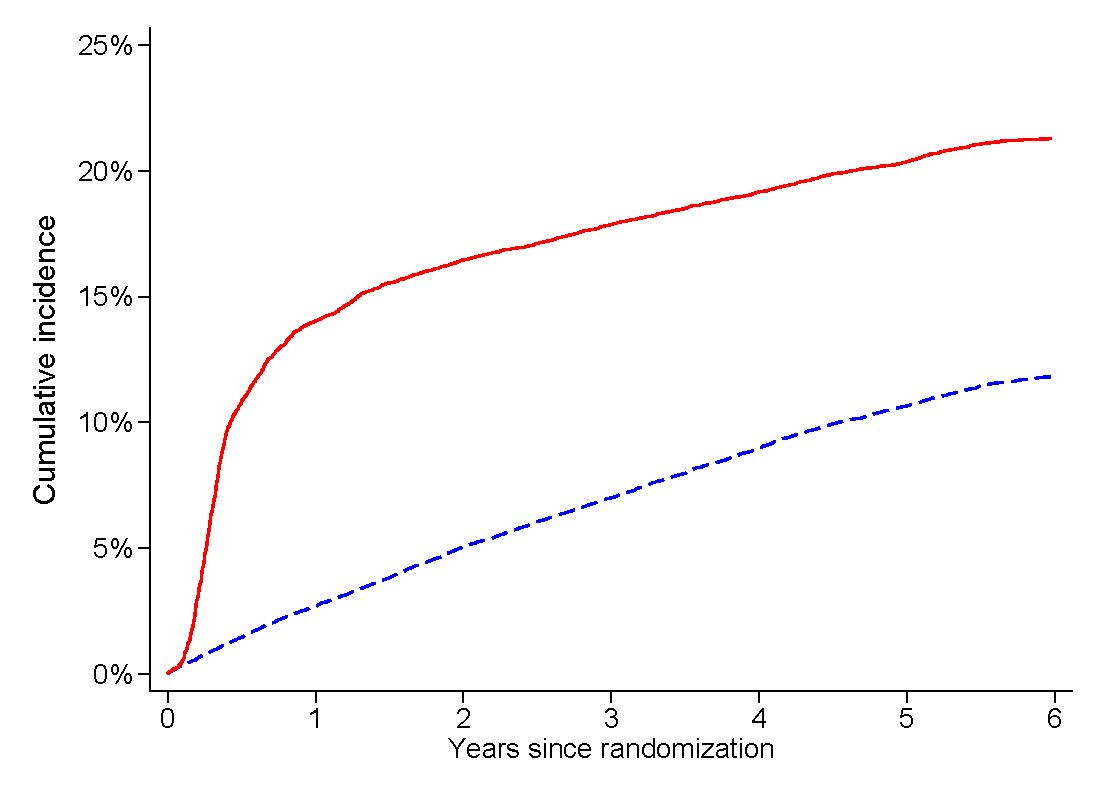

Supplement: S1 Supplementary Material — Fig A. Survey regarding interest in a cardiovascular screening examination—in Danish. Fig B. Hazard ratio by age of the user-defined primary outcome. Fig C. Resulting ROC curve of the inverse probability of treatment weighting model. Fig D. Initiation of antiplatelet (A) and lipid-lowering (B) agents during follow-up. Table A. Definition of outcomes. Table B. Primary and secondary outcomes for probably attendee within the invitation-to-screening and usual-care group. Table C. Distribution of variables before and after inverse probability of treatment weighting. Table D. Initiation of preventive medication. Table E. Adherence to preventive medications. (DOCX) [file pmed.1004403.s001.docx]
