# Supplementary material for: User-defined outcomes of the Danish cardiovascular screening (DANCAVAS) trial: A post hoc analyses of a population-based, randomised controlled trial
Source: PLoS Med. 2024 May 13;21(5):e1004403. doi: 10.1371/journal.pmed.1004403 (PMC11132442; doi:10.1371/journal.pmed.1004403)
Supplement: S1 Statistical Analysis Plan — (PDF) [file pmed.1004403.s003.pdf]

# Statistical Analysis Plan for the Danish Cardiovascular Screening Trial 2

SAP authors: Jes Lindholt[a], Anna Mejldal[b], Rikke Søgaard[c], Axel Diederichsen[d]

Affiliations:
[a] Department of Cardiothoracic and Vascular Surgery, Odense University Hospital, Odense, Denmark
[b] OPEN – Open Patient data Explorative Network, Odense University Hospital and Department of Clinical Research, University of Southern Denmark, Odense, Denmark
[c] Elite Centre CIMA, Clinical Institute, University of Southern Denmark
[d] Department of Cardiology, Odense University Hospital, Odense, Denmark

## Introduction

The primary outcome of the Danish Cardiovascular Screening (DANCAVAS) Trial was presented at the ESC 2022 and simultaneously published in NEJM August 2022. In the primary publication, no adjustments for multiple analyses were performed, so only exploratory analyses of the secondary outcomes were made. Also, we did not include cause-specific mortality, as these data were not available at data extraction in December 2021. The presentation caused global interest and a call for supplemental analyses,- especially regarding the potential benefit in the age group 65-69, and among those not having prior cardiovascular disease (CVD). Another point of criticism was lack of patient involvement, a third point of criticism was lack of a per-protocol analysis to evaluate the effect of the screening examination and intervention without dilution from the non-attenders. As the potential gain for public health could be substantial we have been urged to prolong follow-up and explore the subgroups further.
Consequently, we will now – with help from the target group (patient involvement) – respond to the criticism with new analyses. First, we will present a new primary outcome, a ***patient defined primary outcome***. We aim to send a questionnaire to the target group to define the outcome based upon their preferences. Furthermore, we plan new analyses to evaluate the patient defined primary outcome separate among those without prior CVD and separate in the age-group 65-69 with adjustment for multiple analyses, as well as explore the influence of the screening on the five-year cause-specific mortality rates. In addition, we have planned a per-protocol analyses using use the inverse probability of treatment weighting (IPTW) method to balance the baseline patient characteristics in the control and attendance groups. Finally, we will extract updated outcome data by 31th of December 2022.

## Brief design and material of the DANCAVAS trial

46,611 men, age 64-74 years were randomized 1:2 to a comprehensive imaging- and blood pressure-based screening examination.

## Practical considerations

Screening data were collected using REDCap hosted by OPEN (Open Patient data Explorative Network, Odense University Hospital, Odense Denmark) with project number OP_122. Outcome data, including death, causes of death, hospitalization and medical prescription, were collected from the Danish nationwide registries. Data were analyzed using Stata on OPEN's secure analysis server (OPEN Analyse).

# Planned analyses for the publication: Benefits and harms of the randomized, clinical controlled Danish Cardiovascular Screening (DANCAVAS) trial of 65–74-year-old men

## Inclusion criteria

All men, aged between 65 and 74 years, living in the involved communities at date of randomization.

## Exclusion criteria

There were no exclusion criteria in the study.

## Hypotheses

Three hypotheses are tested:

1. Inviting 65–74-year-old men to a comprehensive CVD screening is associated with benefits preferred by the users, see below
2. Inviting 65-74-year-old men without prior CVD to a comprehensive CVD screening is associated with benefits preferred by the users, see below
3. Inviting 65–69-year-old men to a comprehensive CVD screening is associated with benefit benefits preferred by the users, see below

## Primary outcome

The primary outcome was defined by the target group (user involvement) and based on a questionnaire send to more than 10,000 men (aiming for 1000 responders in each age-group) in January 2023, please see Figure 2. Outcome will be assessed at December 31, 2021 or December 31, 2022.[1]

## Secondary outcomes

- Causes of death (primary and participating) classified as cardiovascular, cancer, other disease, and composite: trauma and suicide (time to event or censoring), assessed at December 31, 2021.
- Composite outcome of major adverse cardiovascular events (MACE); death due to cardiovascular disease, stroke, acute myocardial infarction, heart failure assessed at December 31, 2021.
- Composite outcome of major adverse limb events (MALE); death due to cardiovascular disease, aortic rupture and dissection, critical limb ischemia, and major amputation due to peripheral arterial disease assessed at December 31, 2021.

## Explanatory outcomes

- Stroke; all, ischemic, hemorrhagic, and unspecified after randomization (time to event or censoring), assessed at December 31, 2022.

[1] Cause of death is available until December 31, 2021, while all other variables are available until December 31, 2022.

- Myocardial infarction after randomization (time to event or censoring), assessed at December 31, 2022.
- Heart failure after randomization (time to event or censoring), assessed at December 31, 2022.
- Critical limb ischemia after randomization (time to event or censoring), assessed at December 31, 2022.
- Amputation due to vascular disease after randomization (time to event or censoring), assessed at December 31, 2022.
- Aortic dissection, any site after randomization (time to event or censoring), assessed at December 31, 2022.
- Aortic rupture, any site after randomization (time to event or censoring), assessed at December 31, 2022.
- Attendance rate
- Initiation and adherence to preventive medications after randomization: antithrombotic agents, anticoagulation, lipid-lowering agents, antihypertensive, and antidiabetics
- Elective aortic aneurysm repair after randomization

## Safety outcomes

- Major intracerebral and gastrointestinal bleeding leading to hospitalization after randomization (time to event or censoring), assessed at December 31, 2022
- Cardiac revascularization, peripheral vascular revascularization, and aortic repair after randomization (time to event or censoring), assessed at December 31, 2022
- Incident cancer from 6 months[2] after randomization (time to event or censoring), assessed at December 31, 2022
- Mortality after cardiovascular surgery (30 days)

## Patient preference

Patient preferences were evaluated by a questionnaire send to 10,000 men being 65-74 years old at the time of posting the questionnaire in January 2023. These men were previously (from September 2014 until December 2018) randomized to the control groups in the DANCAVAS I and II trials.

We asked for the main reason for willingness to participate in a CVD screening examination, and those not interested in such screening, were asked about reasons why not (please see Figure 2). With these answers the primary outcome were defined as a composite endpoint of all variables that were preferred by more than half of the users (or target group for the screening examination).

## Sample size considerations

With the use of the PS - Power and Sample Size Program by the Vanderbilt University, (Dupont WD, Plummer WD: 'Power and Sample Size Calculations: A Review and Computer Program', Controlled Clinical Trials 1990; 11:116-28), we calculated the smallest detectable differences to secure that sufficient sample sizes were present:

[2] Incident cancer is registered as a safety outcome to examine if the screening examination and intervention may induce cancer, and as cancer might be an incidental finding in the screening examination, we will blind the first 6 months after randomization.

Hypothesis 1. We are planning a screening study with 16,736 invited men, 29,790 control subjects with no accrual interval, and median follow-up of 7.17 years. Using a significance level of 0.016, and 80% power, the smallest detectable differences in HR are 0.957758 and 1.044918.

Hypothesis 2. We are planning a screening study with 13,169 invited men, 26,338 control subjects with no accrual interval, and median follow-up of 7.17 years. Using a significance level of 0.016, and 80% power, the smallest detectable differences in HR are 0.9523883 and 1.050634.

Hypothesis 3. We are planning a screening study with 9,209 invited men, 18,418 control subjects with no accrual interval, and median follow-up of 7.17 years. Using a significance level of 0.016, and 80% power, the smallest detectable differences in HR are 0.9436017 and 1.060978.

As three hypotheses are tested (overall population, overall population excluding prior CVD and overall population only including the subgroup aged 65-69 years), the adjustment for multiple testing is done as suggested by the Holm-Bonferroni method (https://pubmed.ncbi.nlm.nih.gov/8629727/).

## Statistical principles

All analyses were performed as intention-to-screen and as superiority analyses except the per protocol analysis. The endpoints were compared for the two randomization groups using Cox hazard regression for analyses of unadjusted hazard ratios (95% confidence intervals). Time of randomization defines the onset of risk time and exit from analysis is time of event or censoring on 12-31-2022 whichever came first. Deaths without secondary events are right-censored. Only the first event of each category is counted. Both relative and absolute risk estimates will be reported, as well as the number needed to invite (NNI) in order to save one life will be estimated using Newcombe's method (ref https://pubmed.ncbi.nlm.nih.gov/19519911/).

For the primary analysis of the three hypotheses, two-sided p-values of 0.016 or less are considered to indicate statistical significance. All other analyses are reported with 95% confidence intervals. The widths of the confidence intervals are not adjusted for multiplicity, so the intervals should not be used in place of a hypothesis test.

Preventive medications are reported as counts separately for each group and compared between groups by hazard ratio (95% CI). Individuals who had received a relevant prescription within 1 year before randomization were excluded from analyses. Time of randomization defines the onset of risk time and exit from analysis is time of event or censoring on 12-31-2022 whichever came first. Deaths without events are right-censored.

The model assumption of proportional hazards are assessed with the use of Schoenfeld residuals tests and visual inspection of log-log plots of outcome versus analysis of time. In cases in which the assumption of proportional hazards do not hold, the difference (and 95% confidence interval) in restricted mean survival time at 7 years between participants who underwent screening and those who did not is reported as an alternative to hazard ratios.

In a sensitivity analysis for all outcomes other than death, the competing risk of death is taken into account. Subdistribution hazard ratios and 95% confidence intervals are computed with the use of the method of Fine and Gray.

## Per-protocol analysis

Non-attendance to screening was more than one out of three invited. In order to estimate the benefit of the screening examination, while taking into account that due to selection bias the attending population may differ to the invited and control population, we use the inverse probability of treatment weighting (IPTW) method (Austin PC, Stuart EA. Moving towards best practice when using inverse probability of treatment weighting (IPTW) using the propensity score to estimate causal treatment effects in observational studies. Stat Med. 2015 Dec 10;34(28):3661-79). IPTW uses the propensity score (PS) to balance the baseline patient characteristics in the control and attendance groups by weighting each individual in the analysis by the inverse probability of attending screening.  The propensity score p(X) is the conditional probability of attending screening given pre-screening characteristics. The IPTW is calculated as 1/p(X) for screened individuals, and 1/(1-p(X)) for controls. Both screened individuals with a very low p(X) and controls with a high p(X) have large IPWT's to account for unequal probability of attending screening.

For the per-protocol analyses, the IPTW will be added to all analysis as described above.

## Planned tables and figures and corresponding analyses

### Table 1. Baseline characteristics for all three prespecified hypothesis

Characteristics of the participants will be reported separately for the two randomized groups: invited to screening versus control group, and within the invited to screening group: participants versus non-participants.

| | **All men aged 65-74 years** | | **Men aged 65-74 years without CVD** | | **All men aged 65-69 years** | |
|---|---|---|---|---|---|---|
| **Characteristic** | **Invited to screening (N=XX)** | **Control group (N=XX)** | **Invited to screening (N=XX)** | **Control group (N=XX)** | **Invited to screening (N=XX)** | **Control group (N=XX)** |
| Age – years [numerical] | | | | | | |
| Prescriptions the last year before randomization<br>• Antiplatelet agents – no (%)<br>• Anticoagulants – no (%)<br>• Lipid- lowering agents – no (%)<br>• Antihypertensive agents – no (%)<br>• Antidiabetic agents – no (%) | | | | | | |
| Hospital admission during the last five years before randomization<br>• Stroke – no (%)<br>• Ischemic heart disease* – no (%)<br>• Heart failure – no (%)<br>• Peripheral occlusive arterial disease – no (%)<br>• Aortic aneurysms – No (%) | | | | | | |

Ischemic heart disease: myocardial infarction and coronary revascularization

## Table 2. Primary and secondary outcomes for all three prespecified hypothesis

| **ALL 65-74 YR OLD MEN** | **Invited to screening (N=XX)** | | | **Control group (N=XX)** | | | **HR (95% CI)** | **p value** | **NNI (95%)** |
|---|---|---|---|---|---|---|---|---|---|
| **Outcome** | Events No (%) | Years at risk Median (IQR) | no. of events per 1000 person-years | Events No (%) | Years at risk Median (IQR) | no. of events per 1000 person-years | | | |
| **Primary outcome** | | | | | | | | | |
| Defined by the users | | | | | | | | | |
| **Secondary outcomes** | | | | | | | | | |
| CVD specific mortality | | | | | | | | | |
| Cancer-specific mortality | | | | | | | | | |
| Mortality of other diseases | | | | | | | | | |
| None-disease mortality | | | | | | | | | |
| MACE | | | | | | | | | |
| MALE | | | | | | | | | |
| **MEN WITHOUT PRIOR CVD** | | | | | | | | | |
| **Primary outcome** | | | | | | | | | |
| Defined by the users | | | | | | | | | |
| **Secondary outcomes** | | | | | | | | | |
| CVD specific mortality | | | | | | | | | |
| Cancer-specific mortality | | | | | | | | | |
| Mortality of other diseases | | | | | | | | | |
| None-disease mortality | | | | | | | | | |
| MACE | | | | | | | | | |
| MALE | | | | | | | | | |
| **MEN AGED 65-69 YR** | | | | | | | | | |
| **Primary outcome** | | | | | | | | | |
| Defined by the users | | | | | | | | | |
| **Secondary outcomes** | | | | | | | | | |
| Cardiovascular specific mortality | | | | | | | | | |
| Cancer-specific mortality | | | | | | | | | |
| Mortality of other diseases | | | | | | | | | |
| None-disease mortality | | | | | | | | | |
| MACE | | | | | | | | | |
| MALE | | | | | | | | | |

NNI; number needed to invite. MACE; death due to cardiovascular disease, stroke, acute myocardial infarction, heart failure assessed at December 31, 2021. MALE: death due to cardiovascular disease, aortic rupture and dissection, critical limb ischemia, and major amputation due to peripheral arterial disease assessed at December 31, 2021. None-disease mortality: trauma, homicide, and suicide.

## Table 3. Primary and secondary outcomes for attendee to the screening and probably attendee within the control group specified in the three prespecified hypothesis

| **ALL 65-74 YR OLD MEN** | **Invited to screening (N=XX)** | | | **Control group (N=XX)** | | | **HR (95% CI)** | **p value** | **NNI (95%)** |
|---|---|---|---|---|---|---|---|---|---|
| **Outcome** | Events No (%) | Years at risk Median (IQR) | no. of events per 1000 person -years | Events No (%) | Years at risk Median (IQR) | no. of events per 1000 person -years | | | |
| **Primary outcome** | | | | | | | | | |
| Defined by the users | | | | | | | | | |
| **Secondary outcomes** | | | | | | | | | |
| CVD specific mortality | | | | | | | | | |
| Cancer-specific mortality | | | | | | | | | |
| Mortality of other diseases | | | | | | | | | |
| None-disease mortality | | | | | | | | | |
| MACE | | | | | | | | | |
| MALE | | | | | | | | | |
| **MEN WITHOUT PRIOR CVD** | | | | | | | | | |
| **Primary outcome** | | | | | | | | | |
| Defined by the users | | | | | | | | | |
| **Secondary outcomes** | | | | | | | | | |
| CVD specific mortality | | | | | | | | | |
| Cancer-specific mortality | | | | | | | | | |
| Mortality of other diseases | | | | | | | | | |
| None-disease mortality | | | | | | | | | |
| MACE | | | | | | | | | |
| MALE | | | | | | | | | |
| **MEN AGED 65-69 YR** | | | | | | | | | |
| **Primary outcome** | | | | | | | | | |
| Defined by the users | | | | | | | | | |
| **Secondary outcomes** | | | | | | | | | |
| CVD specific mortality | | | | | | | | | |
| Cancer-specific mortality | | | | | | | | | |
| Mortality of other diseases | | | | | | | | | |
| None-disease mortality | | | | | | | | | |
| MACE | | | | | | | | | |
| MALE | | | | | | | | | |

NNI; number needed to invite. MACE; death due to cardiovascular disease, stroke, acute myocardial infarction, heart failure assessed at December 31, 2021. MALE: death due to cardiovascular disease, aortic rupture and dissection, critical limb ischemia, and major amputation due to peripheral arterial disease assessed at December 31, 2021.

## Table 4. Exploratory outcomes for all three prespecified hypothesis

| **ALL 65-74 YR OLD MEN** | **Invited to screening (N=XX)** | | | **Control group (N=XX)** | | | **HR (95% CI)** | **p value** |
|---|---|---|---|---|---|---|---|---|
| **Event** | Events No (%) | Years at risk Median (IQR) | no. of events per 1000 person-years | Events No (%) | Years at risk Median (IQR) | no. of events per 1000 person-years | | |
| **Cardiovascular diseases** | | | | | | | | |
| Stroke | | | | | | | | |
| - Ischemic | | | | | | | | |
| - Hemorrhagic | | | | | | | | |
| - Unspecified | | | | | | | | |
| Myocardial infarction | | | | | | | | |
| Amputation due to PAD | | | | | | | | |
| Aortic dissection | | | | | | | | |
| Aortic rupture | | | | | | | | |
| Elective aneurysm repair | | | | | | | | |
| **Preventive medication*** | | | | | | | | |
| Initiation of antiplatelet agents | | | | | | | | |
| Initiation of anticoagulants | | | | | | | | |
| Initiation of lipid-lowering agents | | | | | | | | |
| Initiation of hypertensive agents | | | | | | | | |
| Initiation of antidiabetic agents | | | | | | | | |
| **MEN WITHOUT PRIOR CVD** | | | | | | | | |
| **Cardiovascular diseases** | | | | | | | | |
| Stroke | | | | | | | | |
| - Ischemic | | | | | | | | |
| - Hemorrhagic | | | | | | | | |
| - Unspecified | | | | | | | | |
| Myocardial infarction | | | | | | | | |
| Amputation due to PAD | | | | | | | | |
| Aortic dissection | | | | | | | | |
| Aortic rupture | | | | | | | | |
| Elective aneurysm repair | | | | | | | | |
| **Preventive medication*** | | | | | | | | |
| Initiation of antiplatelet agents | | | | | | | | |
| Initiation of anticoagulants | | | | | | | | |
| Initiation of lipid-lowering agents | | | | | | | | |
| Initiation of hypertensive agents | | | | | | | | |
| Initiation of antidiabetic agents | | | | | | | | |
| **MEN AGED 65-69 YR** | | | | | | | | |

| | | | | | | | | |
|---|---|---|---|---|---|---|---|---|
| **Cardiovascular diseases** | | | | | | | | |
| Stroke | | | | | | | | |
| - Ischemic | | | | | | | | |
| - Hemorrhagic | | | | | | | | |
| - Unspecified | | | | | | | | |
| Myocardial infarction | | | | | | | | |
| Amputation due to PAD | | | | | | | | |
| Aortic dissection | | | | | | | | |
| Aortic rupture | | | | | | | | |
| Elective aneurysm repair | | | | | | | | |
| **Preventive medication*** | | | | | | | | |
| Initiation of antiplatelet agents | | | | | | | | |
| Initiation of anticoagulants | | | | | | | | |
| Initiation of lipid-lowering agents | | | | | | | | |
| Initiation of hypertensive agents | | | | | | | | |
| Initiation of antidiabetic agents | | | | | | | | |

* No prescription the last year before randomization. PAD: Peripheral arterial disease

## Table 6. Safety outcomes for all three prespecified hypothesis

| **ALL 65-74 YR OLD MEN** | **Invited to screening (N=XX)** | | | **Control group (N=XX)** | | | **HR (95% CI)** | **p value** |
|---|---|---|---|---|---|---|---|---|
| **Event** | Events – no (%) | Years at risk Median (IQR) | no. of events per 1000 person-years | Events – no (%) | Years at risk Median (IQR) | no. of events per 1000 person-years | | |
| Severe bleeding | | | | | | | | |
| - Intracerebral | | | | | | | | |
| - Gastrointestinal | | | | | | | | |
| Cancer | | | | | | | | |
| Cardiac revascularization | | | | | | | | |
| Lower limb revascularization | | | | | | | | |
| Aortic repair | | | | | | | | |
| 30-d postoperative mortality | | | | | | | | |
| **MEN WITHOUT PRIOR CVD** | | | | | | | | |
| Severe bleeding | | | | | | | | |
| - Intracerebral | | | | | | | | |
| - Gastrointestinal | | | | | | | | |
| Cancer | | | | | | | | |
| Cardiac revascularization | | | | | | | | |
| Lower limb revascularization | | | | | | | | |
| Aortic repair | | | | | | | | |
| 30-d postoperative mortality | | | | | | | | |
| **MEN AGED 65-69 YR** | | | | | | | | |
| Severe bleeding | | | | | | | | |
| - Intracerebral | | | | | | | | |
| - Gastrointestinal | | | | | | | | |
| Cancer | | | | | | | | |
| Cardiac revascularization | | | | | | | | |
| Lower limb revascularization | | | | | | | | |
| Aortic repair | | | | | | | | |
| 30-d postoperative mortality * | | | | | | | | |

*: Deaths within 30 days after surgery coronary or peripheral vascular revascularization, as well aortic repair

## Table 7. Adherence to preventive medications for all three prespecified hypothesis

Adherence to a medication is defined as medication possession ratio (MPR) of at least 80% from 1st redeemed prescription over a time period of 3 years. Values below 80% will be considered as non-adherence. Individuals who failed to redeem prescriptions during the first three years after randomization will not be eligible for analysis, and only individuals who redeemed at least one relevant prescription can be included in the analyses. Five medication groups will be considered: anti-thrombotic agents (A), anticoagulants (B), lipid-lowering agents (C), antihypertensive (D), and antidiabetics (E). The adherence results will be presented as relative risks with 95% confidence intervals.

| Drug class (ATC) | Non-adherent patients in control population (n(non-adherent)/n(total) (%)) | Non-adherent patients in screening population (n(non-adherent)/n(total) (%)) | Relative risk of non-adherence (RR (95%CI)) |
|---|---|---|---|
| **ALL 65-74 YR OLD MEN** | | | |
| Antiplatelet agents (B01AC) | | | |
| Anticoagulants (B01AA, B01AE, B01AF) | | | |
| Lipid-lowering agents (C10) | | | |
| Antihypertensive (C03A, C03B, C07, C08 excl C08DA, C09) | | | |
| **MEN WITHOUT PRIOR CVD** | | | |
| Antiplatelet agents (B01AC) | | | |
| Anticoagulants (B01AA, B01AE, B01AF) | | | |
| Lipid-lowering agents (C10) | | | |
| Antihypertensive (C03A, C03B, C07, C08 excl C08DA, C09) | | | |
| **MEN AGED 65-69 YR** | | | |
| Antiplatelet agents (B01AC) | | | |
| Anticoagulants (B01AA, B01AE, B01AF) | | | |
| Lipid-lowering agents (C10) | | | |
| Antihypertensive (C03A, C03B, C07, C08 excl C08DA, C09) | | | |

## Table 8. Consequences in Quality of Life for all three prespecified hypothesis

Health-related quality of life based on the EQ-5D-3L will be scored using Danish general population-based preference weights in order to generate index values at baseline and for repeated measurements during follow up in the participant-reported outcome (PRO) analysis. The questionnaire was administered to all participants at the screening examination, and electronically questionnaires were send to a random sample of participants in the succeeding years. Additionally, a random sample of non-participants and individuals from the control group received electronically questionnaires

Response rates will be assessed as the proportion of the surveyed, who returned the questionnaire. Completion rates will be assessed as the proportion of responders, who reported a status on all of the five items. Analysis will be based on available data and no imputation will be conducted.

| **EQ-5D-3L** | **Mean difference (95% CI) of change from baseline to first follow-up** | **Mean difference (95% CI) of change from baseline to second follow-up** |
|---|---|---|
| **ALL 65-74 YR OLD MEN** | | |
| **Invited vs controls** | | |
| Profile-based index | | |
| VAS-based index | | |
| **Attenders vs nonattenders** | | |
| Profile-based index | | |
| VAS-based index | | |
| **Positive vs negative test** | | |
| Profile-based index | | |
| VAS-based index | | |
| **MEN WITHOUT PRIOR CVD** | | |
| **Invited vs controls** | | |
| Profile-based index | | |
| VAS-based index | | |
| **Attenders vs nonattenders** | | |
| Profile-based index | | |
| VAS-based index | | |
| **Positive vs negative test** | | |
| Profile-based index | | |
| VAS-based index | | |
| **MEN AGED 65-69 YR** | | |
| **Invited vs controls** | | |
| Profile-based index | | |
| VAS-based index | | |
| **Attenders vs nonattenders** | | |
| Profile-based index | | |
| VAS-based index | | |
| **Positive vs negative test** | | |
| Profile-based index | | |
| VAS-based index | | |

EQ-5D-3L, EuroQol 5-dimension 3-level; CI, confidence interval; LS, least squares. VAS, visual analogue scale

Figure 1. Enrollment, Randomization, and Follow-up.

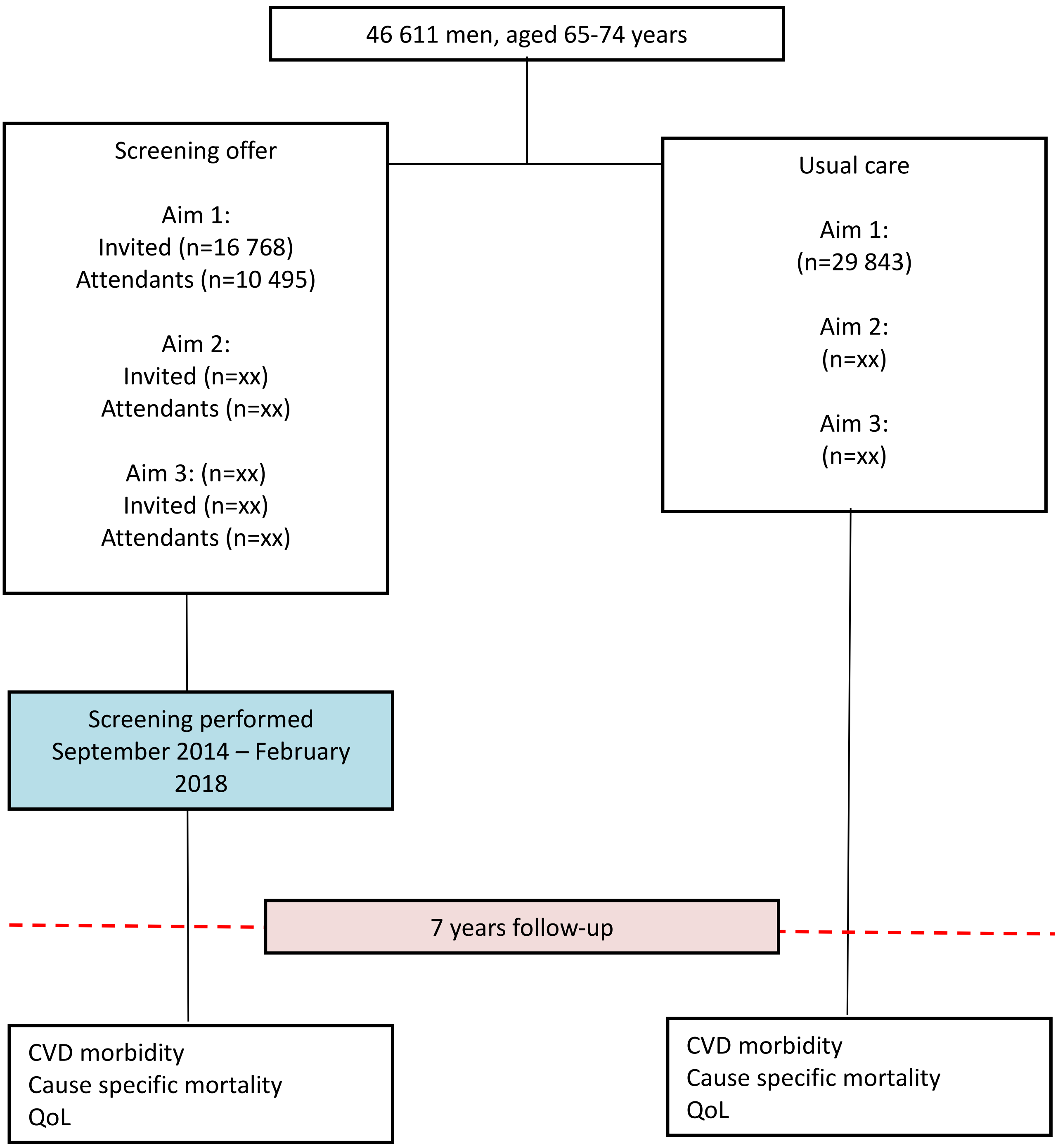

## Figure 2. Questionnaire regarding users' interest in cardiovascular screening

Patient preferences regarding a cardiovascular screening examination were evaluated by a questionnaire send to 10,000 men 65-74 years old at the time of posting the electronic questionnaire. They were asked about their interest in a screening examination. If they choose to have a screening examination they were asked why. Finally, the primary endpoint were composed by all reasons chosen by more than 50% of the respondents. Additionally, if they were not interested in such screening, they were asked why not.

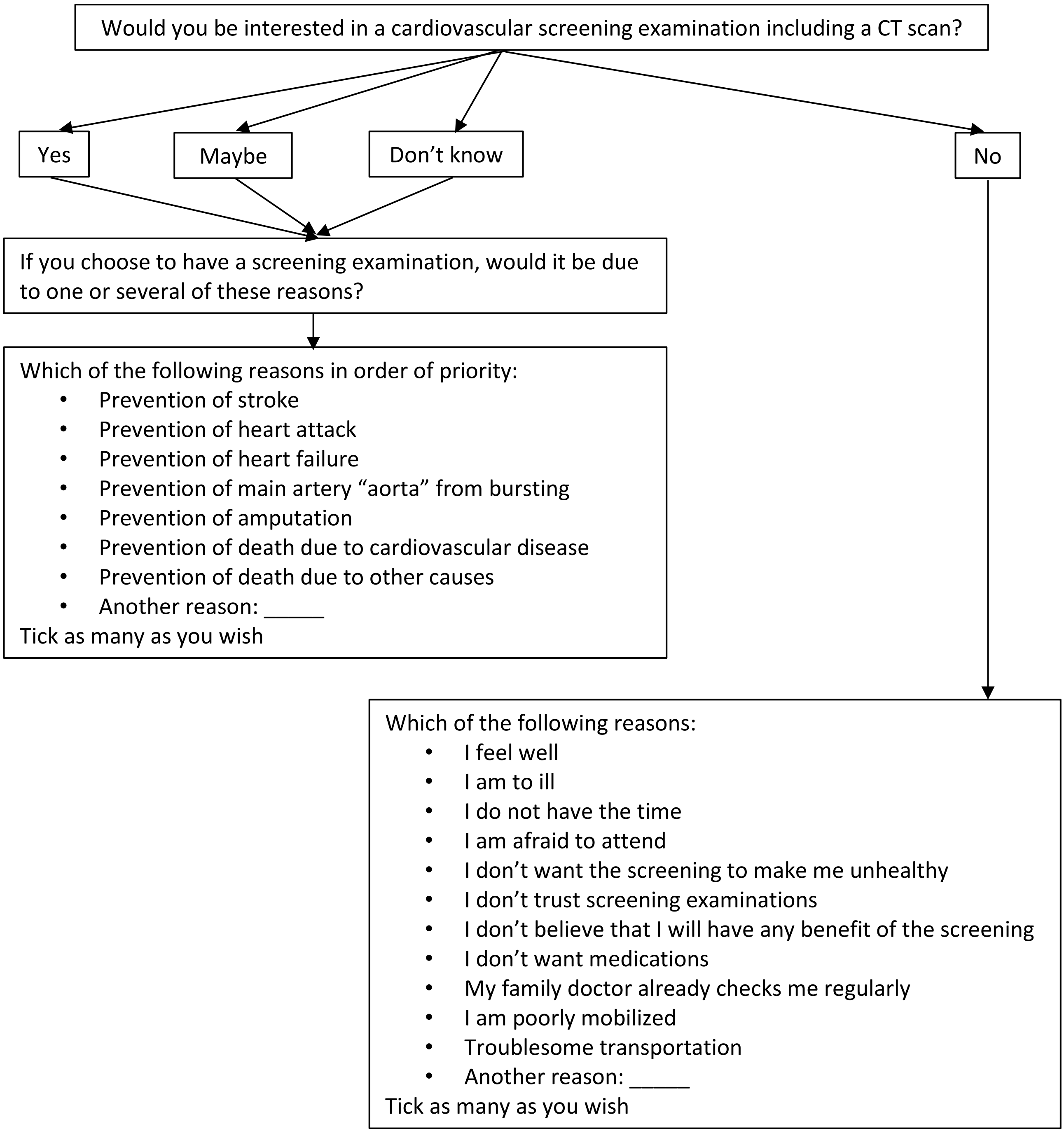

## Figure 3. Effects of the DANCAVAS screening on primary and secondary outcomes for all three prespecified hypothesis.

Cumulative event curves from the two randomized groups will be generated with the use of the Nelson-Aalen cumulative hazard estimates. The primary outcome as defined by users will be shown in Panel A, while the secondary outcomes (Cardiovascular specific mortality (B), Cancer-specific mortality (C), Other diseases specific mortality (D), Trauma and suicide- specific mortality (E) Major adverse cardiovascular events (MACE) (F) and Major adverse limb events (MALE) (G)) will be shown separately in Panel B-G.

## Figure 4. Initiation of preventive actions for all three prespecified hypothesis

Six plots demonstrating initiation of preventive actions in the two randomized groups will be generated. Baseline numbers will be individuals who have not redeemed a prescription for anti-thrombotic agents (A), anticoagulants (B), lipid-lowering agents (C), antihypertensive (D), and antidiabetics (E), respectively, the last year before randomization. Panel F illustrates elective aortic aneurysm repair.

## Figure 5. Relation between age and screening on the patient defined primary outcome

The y-axis will show the hazard ratio of the patient defined primary outcome in the invited group compared to the non-invited group. Hazard ratios will be estimated for each of the sequentially overlapping subpopulations created by a sliding windows approach iterating through increasing age in 3-year age groups. These hazard ratios will be plotted as a circle, with the median age of the group as the x-axis. A colored area will describe the exact 95% confidence intervals: Based on the estimated hazard ratios and corresponding median ages, a polynomial, moving-average regression plot with locally estimated smoothing will be constructed.
