# Supplementary material for: User-defined outcomes of the Danish cardiovascular screening (DANCAVAS) trial: A post hoc analyses of a population-based, randomised controlled trial
Source: PLoS Med. 2024 May 13;21(5):e1004403. doi: 10.1371/journal.pmed.1004403 (PMC11132442; doi:10.1371/journal.pmed.1004403)
Supplement: Attachment — Submitted filename: Reply to Editors and reviewers .docx [file pmed.1004403.s004.docx]

-----------------------------------------------------------

Editorial comments:

1. We really appreciate the premise of your study, and we agree with the reviewers that the “user-defined” outcome is innovative. However, we have some concerns regarding the potential for introducing unintended bias given the approach taken to determine the outcome. Please include a copy of the survey that was sent to the participants to complete as supporting information when you re-submit your manuscript. Further, when considering that the primary trial demonstrated a significant reduction in MACE, we have some concerns regarding novelty of the outcome data presented here.

*Response: Thank you for these nice comments.*

*The questionnaire was send and responded online. We have added the Danish version of the questions in the supplement (Supplementary Figure 1). The questions are translated to English and displayed in Figure 2 in the manuscript.*

*In our NEJM publication, we as investigators defined the primary outcome to be all cause mortality, while a secondary composite outcome was all-cause death, stroke and myocardial infarction. The secondary outcome was hypothesis generating, and no p-values were given. In this manuscript, the primary outcome was defined by the target group prior data collection. The majority requested the outcome to be stroke and myocardial infarction – not including death. Regarding the novelty, we believe that both the involvement of the target group and the outcome are original. Especially that avoiding death wasn’t the primary wish from the users, rather avoiding disease and disability. It is discussed in the discussion (Section ”Definition of a primary outcome”, page 14 line 13-17).*

2. Please be explicit in the title and the abstract that is a post-hoc sub-study/follow-on study of the primary trial. We suggest reporting in-line with CONSORT explicitly stating the sub-study nature and ensuring that the abstract details the main trial items in 2-3 sentences, including the study population, dates, intervention and primary outcome. The majority of the abstract should then describe the complete details of this sub-study. Please complete the CONSORT checklist and ensure that all components of CONSORT are present in the manuscript as well as clearly defined details of this sub-study. When completing the checklist, please use section and paragraph numbers, rather than page numbers as these often change in the event of publication.

*Response: We have added “post-hoc” in the title and abstract.*

*The abstract now meet the demands of CONSORT, including study population, dates, intervention and primary outcome, and describe the complete details of this sub-study.*

*CONSORT checklist is added.*

*.*

3. We agree with the academic editor (please see below) that there is a lack of clarity regarding how participants were recruited for this sub-study and request that additional details are included. We think that the respondents were an entirely different group of men but please clarify and be explicit in your methods.

*Response: We acknowledge and have clarified in the section “Survey to the target group” (page 8, line 17-22).*

4. You refer to ‘prespecified secondary outcomes’ in your methods section including MACE which, as noted above, have already been reported in the primary trial as such we are confused about the use of ‘prespecified’ here. Are these the same secondary outcomes as the detailed in the primary trial? Please clarify.

*Response: We acknowledge, this is confusing. Both the user-defined primary outcome and the secondary outcomes in this manuscript were described* in the *statistical analysis plan (published January 17, 2023,* [*https://www.isrctn.com/ISRCTN12157806*](https://www.isrctn.com/ISRCTN12157806)*). However, most important is that the user-defined primary outcome was prespecified. Accordingly, we have deleted “prespecified” from the “Outcome” section (page 9, line 10), and added “prespecified” in the description of the primary endpoint in the “Survey to the target group” section (page 9, line 3-4).*

-----------------------------------------------------------

Comments from the reviewers:

Reviewer #1: This is a well conducted and well written study and the topic is both interesting and innovative in its use of a "user defined" end-point. The paper is given extra importance that the overall endpoint of the original study was neutral but the screening programme did reduce the combined end-point of MI and stroke, as chosen by the target population. This is very useful data to inform the design of potential future CV screening programmes. The fact that the adverse event of intra-cranial bleeding was also increased gives particular importance to the debate as to whether aspirin should be included in primary prevention programmes - even if screening has documented asymptomatic vascular disease. The authors do discuss this, but perhaps this section could be somewhat expanded given that there is real disagreement in different guidelines around the use of aspirin, although all guidelines concur on the use of statins.

*Response: Thank you.*

*We included the ACC/AHA and the ESC guidelines from 2019 and 2021, respectively, in first version the manuscript, but in this version we have included the new NICE guidelines from 2023. Due to lack of randomized trials, the NICE guidelines do not recommend aspirin – even if CAC is documented (page 15, line 17-19).*

Reviewer #2: This is an excellent piece of work examining the preferred patient outcome for CV screening and then the effects of screening with CT, ABBP and bloods on this outcome in Danish males. The effects of aspirin are salutary but those of statins seem impressive

*Response: Thank you.*

I have very few comments.

1. The assertion in line 2 that 'cardiovascular diseases remain the most frequent cause of death and is associated with a reduction in disability-adjusted life years.1' needs qualifying. In many parts of the world this is not the case and in some parts of Europe cancer is now more common. Perhaps 'cardiovascular diseases remain the most frequent cause of death in adults living in middle and high income countries'?

*Response: The statement is in accordance with the reference (European Society of Cardiology: cardiovascular disease statistics 2021. Eur Heart J 2022; 43: 716-99). However, it is noted that there is a transition towards cancer becoming the most common cause of death in a number of high-income countries as well. Accordingly, we have changed as suggested (page 6, line 2-3).*

2. At the end of the introduction the last sentence is unclear. 'In this study, we aimed to assess whether the comprehensive DANCAVAS screening is requested and leads to a reduction in a user-defined outcome'. Perhaps ..'aimed to assess the community interest in CV screening, patient preferred outcomes for such screening and the effects of screening on these outcomes.

*Response: Thank you for this proposal. It is surely an improvement, and we have changed as suggested (page 6, line 19-21).*

3. 'e-Boks' page 8 requires explanation for non Danish readers

*Response: Please see our response to the Academic Editor (2. Comment)*

4. 'at the time of postal' should read 'at the time of posting'.

*Response: Thank you. We have changed the first paragraph in the section “Survey to the target group” (page 8, line 18-19).*

5. On page 12 Prevention of stroke and myocardial infarction was selected by 4,593 (50.5%) and 4,948 (54.4%), respectively, as the primary motivation for participating in the screening examination' underestimates the popularity of the end points. % Figures for those replying would be equally valuable and give a better insight in to the popularity of the end point. These could easily be added. The views on the preferred end point of those who didnt reply or who were not interested in screening anyway are of limited interest.

*Response: We acknowledge the comment, but we sought to understand the wish by the entire target group, and not just those who attended. Therefore, we defined the primary outcome in advance as a composite endpoint of all variables preferred by more than half of the target group.*

6. The HRs for the outcomes are heavily dependent on the care offered as 'usual care'. Can the authors provide any data on how well or how badly CV risk is addressed in Danish primary care? Are men of this age routinely offered risk assessment using Q-risk or similar Framingham type assessments of risk? What is the ppn of Danish men of this age being treated with cholesterol and BP lowering drugs? The better this 'usual' care, the more difficult it will be to detect the effect of screening...

*Response: This is another relevant comment. In Denmark, risk assessment, including measurement of blood pressure, lipids and HgbA1c, at the primary physician is a free and common service. As displayed in Table 1, Danish elderly men is very frequently treated with lipid-lowering (37%) and antihypertensive (52%) agents. Thus, cardiovascular risk factors seems to be very well treated among Danish elderly men. Against this background, the results seems quite impressive. This is added in the section “Randomisation, screening program and intervention in the DANCAVAS trial” (page 7, line 24 to page 8, line 1-3).*

7. How were men with existing CVD handled? Were they included in DANCANVAS in which case the effects of the intervention will be diluted.

*Response: Men with prior CVD were not excluded, and this diluted the intention-to-screen analyses. We did perform a sensitivity analyses, and the benefit of the screening examination was amplified among men without prior CVD (HR 0.93 versus 0.89). Due to complexity this is not included in the paper, but briefly acknowledge in the Limitation section (page 17, line 3-4).*

Reviewer #3: Alex McConnachie, Statistical Review

Diederichsen et al present a secondary analysis of the DANCAVAS trial in which men were randomly assigned to receive the offer of a detailed cardiovascular screen or not. This paper is looking at a composite outcome of incident MI or stroke, since these two outcomes were selected as being of most importance to the target population. The choice of primary outcome is a clear strength of this paper, giving primacy to the outcomes that patients themselves are most keen to see prevented. This review considers the use of statistics in the paper.

Generally, I think these are very good. Main analyses are done on an intention to screen basis; there is a separate per-protocol analysis, though not a naive analysis, but done using inverse probability weighting. Outcomes are compared using Cox regression (with the proportional hazards assumption appropriately checked), and the number needed to invite (NNI) for screening to prevent one event is reported. Various secondary and explanatory outcomes are reported, in addition to the patient-selected primary outcome. I do have a few comments, but these are all quite minor.

1. The analyses use Cox models without adjustment, though one could argue that analyses should adjust for geographic region, since this was included as a stratification variable in the randomisation.

*Response: We appreciate your suggestion regarding the inclusion of the geographical region variable. Unfortunately, this variable has not been uploaded to Statistics Denmark where we analyze our data, and this process is very time-consuming and would cause some delay. While we acknowledge the importance of taking into account the stratifying variables in randomized controlled trials, we would like to highlight that the region of Southern Denmark included in our study is notably homogenous, mirroring the overall homogeneity of the country. Consequently, we believe that the absence of this geographical region does not significantly impact the validity and generalizability of our findings. Nevertheless, we understand the importance of thorough analysis and plan to incorporate the suggested variable in future studies once it becomes available through Statistics Denmark.*

2. The statistical methods say that both relative and absolute risk estimates are reported, though I did not see anything in the results in terms of absolute risk differences. I think this would help when judging the benefit of screening in relation to the primary outcome, versus the increase in intracranial bleeding. The NNI is related to absolute risk differences, but it is not clear over what time frame these NNI estimates relate to. Note, the methods section talks of the NNI to save one life, even though this paper is reporting mainly non-fatal events as outcomes.

*Response: Thank you for the comment. We have added absolute risk differences, and noted that the NNI is calculated at 6 years (Table 2; page 3, line 22; page 10, line 17; page 12, line 17).*

3. There is also mention of RMST analysis in the methods section, but again, I saw nothing in the results about this. RMST was to be used if there was evidence of non-PH in the Cox models. Perhaps this never occurred? If so, maybe this does not need to be mentioned in the methods section. I do not recall seeing anything about the PH assumption in the results. Perhaps this could be clarified.

*Response: Sorry, we included this in the statistics section prior to analyzing the data as the RMST analysis was in case of the models not fulfilling the PH assumption. However, none of our models failed the PH assumption. The statistical and results section are now changed accordingly.*

4. This IPTW analysis is good to see, though the reference provided (number 10) is for the use of IPTW in observational studies. The only good reference I have found on the use of IPTW in randomised trials is Jo and Stuart (2009) (<https://doi.org/10.1002/sim.3669>), which describes how to estimate the CACE (complier average causal effect), which I think is what is being estimated in this study. They suggest using the odds of compliance (screening) to weight the control group, rather than the inverse probability of not being screened, which I believe is the same as using stabilised weights in standard IPTW, which may be slightly better. Also, in Jo and Stuart (2009), it is clear that non-compliers in the intervention group are excluded from the analysis (given a weight of zero) - that is not explicitly stated in the current paper, though I believe it was done.

*Response:*

*Thank you for the suggestion. We have changed to using the odds of compliance only on controls, and a weight of 1 on compliers as suggested. We have also added your suggested reference, and clarified in the statistics section (page 11, line 4 and line 8-11; Supplementary Table 2).*

*It is correct that we excluded non-compliers in the intervention group in our analysis.*

Reviewer #4: Thank you for this very important study, which is part of the larger DANCAVAS study. The manuscript is written in a clear, concise and informative manner. I have no major comments to make, but I just wanted to ask whether your data would allow you to identify or suggest ways to adjust for the slightly increasing risk of intracranial haemorrhage with intensive medical prophylaxis (which is what you have found in this post-hoc analysis)? However, I realise that this is probably beyond the scope of the paper presenting the results of this study, and I congratulate you on your important work.

*Response:*

*Thank you. We understand the reviewers’ idea behind this suggestion, but in discussion we could not come up with a analysis that would fit the scope of the paper and properly adjust for the increased risk of intracranial haemorrhage. We will however keep this in mind for future studies, or future publications on these data.*

-----------------------------------------------------------

Comments from the academic editor:

I am familiar with the DANCAVAS trial. A few comments and questions:

1. The authors write that the user-defined outcome "was defined as a composite of reasons chosen by more than 50% of the population." Was this prespecified before the survey was done or post-doc? I think this is important to describe to readers because it was already known from DANCAVAS that the composite from stroke/MI/death has a p-value of <0.05. So there could have been an unintentional temptation ("researcher degrees of freedom) to develop a user outcome that was a "significant" result.

*Response: Yes, the definition of the user-defined outcome was prespecified in the statistical analysis plan (published January 17, 2023,* [*https://www.isrctn.com/ISRCTN12157806*](https://www.isrctn.com/ISRCTN12157806)*) before the survey was done. This added in the section “Survey to the target group” (p 9, line 3-4).*

2. I would like more details on sampling of the target group. Were the men sampled using the same eligibility criteria and underlying registry from which the original participants were sampled? What is e-Boks? How do they know the men were not aware of DANCAVAS? What were the characteristics of men invited in the survey and how did those who participated differ from those who did not participate?

*Response: We apologies for the confusion. The men in the target group was sampled from the Danish registries applying the same criteria as the original participants; men aged 65 to 74 years. We can’t tell for sure, that the target group were unaware about the DANCAVAS trial, but DANCAVAS has not been discussed in the public, and we did not explicit mention the DANCAVAS trial in introduction to the survey. This is clarified in the section “Survey to the target group” (page 8, line 18-20).*

*“E-boks” is an online mailbox used by Denmark's municipalities and state authorities. This is briefly mentioned in the section “Randomisation, screening program and intervention in the DANCAVAS trial” (page 7, line 21-22).*

*Unfortunately, we have no further characteristics from the target group than age and sex.*

3. In addition to the IPTW analysis, I wonder if a useful complementary method to get at the effect size of screening itself (rather than the invitation to screen) would be to use an instrumental variable approach. Some experts use the phrase "Contamination adjusted intention to treat" for this approach; see the below citation. This isn't a requirement for me for publication, but I do wonder if it would strengthen the paper in giving clinicians an answer to the question that is most directly relevant to them, i.e., what is the effect size and NNT of screening for this patient in front of me.

Sussman JB, et al. BMJ 2010;340:c2073.

Cuzick J, et al. Statistics in Medicine 1997;16:1017-29.

This approach has been used a several important screening/invitation trials recently, for example, the recent colonoscopy trial in NEJM:

Bretthauer M, et al. N Engl J Med 2022;387:1547-56.

*Response:*

*Thank you for your feedback. Upon reviewing the literature, we observed that the classic "Contamination Adjusted Intention-to-Treat" (CA ITT) approach is predominantly employed in studies with binary outcomes. We could not find references detailing its implementation in a time-to-event setting.*

*Your latest reference, which analyzes time-to-event data, appears to incorporate adjustments for confounders in their CA ITT approach. This aligns closely with our methodology, where we employ weights. Furthermore, this reference (https://www.ncbi.nlm.nih.gov/pmc/articles/PMC3230230/) draws an equivalence between the CA ITT method and the Complier Average Causal Effect (CACE), and as the statistical reviewer points out, our method is equivalent to the CACE method.*

*We are open to further discussion or clarification if needed.*

4. One thing that gives me pause is how the user-centered outcome was chosen.

The justification and methods feel very slender. There are lots of different consensus and deliberation methods to work with patients; why not choose one of those methods? Why are no other studies cited that build a background texture for this approach (i.e., like a formative paper or something, even an a priori protocol). How did theory guide this? It just feels a bit ad hoc as written currently.

This user-centered outcome is really the thing that advances science, yet there are minimal details on this. The outcome data has essentially already been published in the NEJM where MI/CVD/death was already reported as a "significant" secondary outcome (p. 7 of the NEJM paper).

*Response: We acknowledge this comment, and try to clarify.*

*First: We find the outcome novel. Importantly, avoiding death wasn’t the primary wish from the users, rather avoiding disease and disability.*

*Second: The DANCAVAS publication drew a global interest, but also criticism regarding the lack of patient involvement. However, we did involve patients in the past. After initiation the trial, we were concerned about the participation rate, and explored this in two publications (Heart. 2019 May;105(10):761-767 and Patient. 2019 Oct;12(5):491-501). Unfortunately, these publications remained largely theoretical. Due to completion of the inclusion, we were unable to involve users in the planning of the study. Instead, we sought to explore and understand what outcome the target group requested. Thus, in this manuscript, our aim was to provide more specific details about patient expected benefits to a cardiovascular screening examination, please see the associated a priori published statistical analysis plan (https://www.isrctn.com/ISRCTN12157806). We sought to ask randomly selected men aged 65-74, who had not been invited to participate in the DANCAVAS trial previously, regarding their interest in a cardiovascular screening examination, and why they were interested. To achieve this, we sent a questionnaire to 10,000 men, and predetermined the outcome based upon their preferences.*
